# Unilateral hearing during development: hemispheric specificity in plastic reorganizations

**DOI:** 10.3389/fnsys.2013.00093

**Published:** 2013-11-27

**Authors:** Andrej Kral, Silvia Heid, Peter Hubka, Jochen Tillein

**Affiliations:** ^1^Cluster of Excellence, Department of Experimental Otology, Institute of Audioneurotechnology, ENT Clinics, Hannover Medical SchoolHannover, Germany; ^2^Department of Physiology and Otolaryngology, J. W. Goethe UniversityFrankfurt am Main, Germany

**Keywords:** cochlear implant, plasticity, single-sided deafness, critical periods, development

## Abstract

The present study investigates the hemispheric contributions of neuronal reorganization following early single-sided hearing (unilateral deafness). The experiments were performed on ten cats from our colony of deaf white cats. Two were identified in early hearing screening as unilaterally congenitally deaf. The remaining eight were bilaterally congenitally deaf, unilaterally implanted at different ages with a cochlear implant. Implanted animals were chronically stimulated using a single-channel portable signal processor for two to five months. Microelectrode recordings were performed at the primary auditory cortex under stimulation at the hearing and deaf ear with bilateral cochlear implants. Local field potentials (LFPs) were compared at the cortex ipsilateral and contralateral to the hearing ear. The focus of the study was on the morphology and the onset latency of the LFPs. With respect to morphology of LFPs, pronounced hemisphere-specific effects were observed. Morphology of amplitude-normalized LFPs for stimulation of the deaf and the hearing ear was similar for responses recorded at the same hemisphere. However, when comparisons were performed between the hemispheres, the morphology was more dissimilar even though the same ear was stimulated. This demonstrates hemispheric specificity of some cortical adaptations irrespective of the ear stimulated. The results suggest a specific adaptation process at the hemisphere ipsilateral to the hearing ear, involving specific (down-regulated inhibitory) mechanisms not found in the contralateral hemisphere. Finally, onset latencies revealed that the sensitive period for the cortex ipsilateral to the hearing ear is shorter than that for the contralateral cortex. Unilateral hearing experience leads to a functionally-asymmetric brain with different neuronal reorganizations and different sensitive periods involved.

## Introduction

In developmental manipulations of the symmetry of auditory input, as occurs with unilateral deafness (Reale et al., 1987; Vale et al., 2004; Langers et al., 2005; Burton et al., 2012; Kral et al., 2013) or asymmetric moderate hearing loss (King et al., 2001; Popescu and Polley, 2010), the hemispheres can be differentiated in respect of the anatomical relationship to the (better) hearing ear. Plastic reorganizations are often reported in the hemisphere contralateral to the hearing ear. However, the ipsilateral cortex also receives asymmetric input and likely participates in behavioral consequences of unilateral hearing. The present study investigates whether the function of the primary field A1 of the ipsilateral and the contralateral cortex differs in unilateral deafness.

The primary auditory cortex contains mainly binaural neurons—neurons responsive to stimulation of only one ear are virtually absent (Zhang et al., 2004). Many different aural interaction patterns have been described in neuronal recordings, even in the same neurons, depending on the exact binaural temporal and intensity relations (Zhang et al., 2004). The majority of auditory neurons has receptive fields covering large portions of the contralateral hemifield (Middlebrooks et al., 1994). Stimulation of one ear most frequently leads to excitation in the neurons of the contralateral cortex but may cause excitation or inhibition in the ipsilateral cortex (Imig and Adrián, 1977). Because of the stronger responses at the contralateral cortex, and because of the shorter latency of the responses at the contralateral cortex, the term “aural dominance” (Imig and Adrián, 1977) or aural preference (Kral et al., 2013) has been introduced. Contralateral “dominance” is the consequence of the cortical representation of the contralateral acoustic hemifield.

Recently, effects of unilateral deafness have attracted clinical interest owing to the predominantly monaural therapy of prelingual deafness with one cochlear implant (Graham et al., 2009; Gordon et al., 2013) and the relatively high incidence of unilateral deafness (Eiserman et al., 2008; Watkin and Baldwin, 2012). Unilateral deafness is now considered an indication for cochlear implantation of the deaf ear, but so far mainly in cases of postlingual deafness due to tinnitus in the deaf ear (Vermeire et al., 2008; Buechner et al., 2010; Firszt et al., 2012). The effects of congenital or early onset of unilateral hearing are less well explored.

Many previous studies have investigated the plasticity of the brain following cochlear implantation and have described, both in humans and in an animal model, sensitive developmental periods for plasticity (review in Kral and Sharma, 2012). When hearing is imbalanced (as caused by early unilateral hearing loss following periods of binaural hearing), the auditory system reorganizes (Bilecen et al., 2000; King et al., 2001; Langers et al., 2005; Popescu and Polley, 2010; Kral et al., 2013). Central reorganizations have been suggested previously in cases including sequential cochlear implantations in children (Peters et al., 2007; Graham et al., 2009; Gordon et al., 2013; Illg et al., 2013). The outcomes for the implantation of the second ear critically depend on the age at implantation of the first ear and the delay between the implantations (Sharma et al., 2007; Graham et al., 2009; Illg et al., 2013). Recently, two studies elucidated the mechanisms behind such phenomena. In the first study, a sensitive period for the reorganization of aural preference at the cortex ipsilateral to the first implanted ear has been demonstrated using electrophysiological methods in cats (Kral et al., 2013). This study uncovered the mechanism behind the critical dependence of the outcome of the second implantation on age at first implantation. The second study confirmed and extended the findings using electroencephalographic recordings in sequentially-implanted children (Gordon et al., 2013).

**Figure 1:**
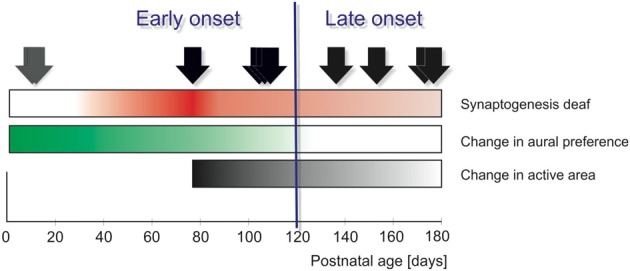
**Onset of unilateral hearing in ten animals (arrows) compared with the developmental time-line of deaf cat auditory system as reflected in the auditory cortex**. The intensity of the color represents the extent of the given change. “Functional synaptogenesis” (development of synaptic function, measured by current source density analyses of evoked responses in the cortex, thus reflecting synaptic counts combined with synaptic function) in binaurally deaf animals has been shown to be delayed by 1.5 months compared to that in hearing animals, with a maximum of evoked synaptic currents around 3 months (Kral et al., 2005, red). Previous studies in congenitally deaf cats demonstrated two sensitive periods: one for reorganization reflected at cortex ipsilateral to hearing ear (green), terminating between 3.5 and 4.2 months (Kral et al., 2013), the other sensitive period for increase in activated volume of tissue (“active area”) in the contralateral field A1 (and the corresponding response latencies; black), terminating between 5 and 6 months (Kral et al., 2002; Kral and Sharma, 2012). In the present study, distinction between early and late onset was based on sensitive period of aural preference (Kral et al., 2013), whereas sensitive period for expansion of the active area extends beyond this timeframe. Gray arrows: unilaterally congenitally deaf animals; black arrows: binaurally deaf animals, implanted with a unilateral cochlear implant at different postnatal times.

The present study directly compares the hemispheric effects of unilateral hearing. Congenitally deaf (white) cats were selected from a colony of deaf white cats using an early hearing screening procedure described earlier (Heid et al., 1998). Some animals were implanted with a cochlear implant during early development, so that stimulation onset was possible from 2.5 to 6 months of age, covering the age of synaptic development of deaf cats (Figure 1, Kral and Sharma, 2012). Two animals were born unilaterally deaf (Kral et al., 2013) and were used as models of very early asymmetric hearing (single-sided deafness).

The present study demonstrates that reorganizations following unilateral hearing (deafness) show a specificity for the hemisphere. The cortex ipsilateral to the hearing ear demonstrates a functional shift toward the hearing ear (Kral et al., 2013). On the other hand, at the hemisphere contralateral to the hearing ear a reduction of the responses to the deaf ear was observed. Despite some ear-specific effects in onset latency, the morphology of the local field potentials (LFPs) showed more pronounced differences between hemispheres than differences observed on the same hemisphere when comparing the responses to stimulation of the deaf and the hearing ear. This demonstrates hemispheric specificity of the reorganizations. Finally, reorganizations observed in onset latency demonstrated a shorter sensitive period for plasticity in the hemisphere ipsilateral to the hearing ear when compared to the contralateral hemisphere.

## Materials and methods

The present experiments complement a previous study and the methods are described in detail there (Kral et al., 2013). Here, we summarize the most important technical aspects of the method.

### Animals

Experiments were performed on 10 cats. In all animals, hearing was strongly asymmetric (Table 1): two animals had normal hearing in one ear (hearing thresholds being <40 dB SPL) and were unilaterally congenitally deaf in the other ear (hearing thresholds >110 dB SPL). The remaining animals were binaurally congenitally deaf cats (CDCs) monaurally implanted with a custom-made cochlear implant at the age of 2.5–6.0 months and subjected to chronic electrostimulation. The implantation ages of unilateral animals are given in Table 1. Not all stimulus/recording combinations could be investigated in each animal.

**Table 1:** **Implantations, age at final experiments and investigated cortex for the animals in the study**.

**Animal**	**Age at onset of unilateral hearing [months]**	**Age at experiment [months]**	**Contralateral cortex**	**Ipsilateral cortex**
1	Congenital	>12		•
2	Congenital	>12	•	•
3	2.5	4.5	•	•
4	3.5	9	•	•
5	3.5	6.5	•	
6	3.5	5.5	•	
7	4.2	9.2		•
8	5.0	10	•	•
9	6.0	11	•	•
10	6.0	8	•	•

All animals obtained from our colony of deaf white cats underwent hearing screening within the fourth week of life. The screening procedure was based on a longitudinal study of hearing in deaf white cats recorded every two days after birth and is described in detail elsewhere (Heid et al., 1998).

All experiments were approved by the local state authorities and were performed in compliance with the guidelines of the European Community for the care and use of laboratory animals (EU VD 86/609/EEC) and the German Animal Welfare Act (TierSchG).

To investigate developmental plasticity in animals with unilateral hearing, chronic stimulation by a cochlear implant was initiated at two different ages, reflecting the results of previous studies (Figure 1):

- *early* (2.5 and 3.5 months, early implanted animals), when the naive cortex shows a developmental peak in evoked synaptic activity (Kral et al., 2005) and the animals showed plasticity of aural preference (Kral et al., 2013),
- *late* (after 4.0 months), when synaptic activity in deaf animals fell below the level of hearing controls and the known sensitive period for aural preference has already expired (Kral et al., 2013).

### Implantation and chronic stimulation

Implantations were performed under sterile conditions in anesthetized animals as described previously (Kral et al., 2002). Animals were premedicated with 0.25 mg atropine i.p. and anaesthetized with ketamin hydrochloride (24.5 mg/kg Ketavet, Parker-Davis, Germany) and xylazine hydrochloride (1 mg/kg, Bayer, Germany) with supplementary doses when necessary. The animal's status was monitored by capnometry, electroencephalography, electrocardiogram and pulse oximetry. The bulla was exposed via a retrocochlear cutaneous incision and a subsequent soft separation of the muscles overlying it. The bulla was opened using a drill. The membrane of the round window was carefully removed with a sharp hook and the electrode carrier was implanted through the round window. The implant was fixed using a suture (non-absorbable thread) at the dorsal thickened part of the bulla. The bulla was tightly closed using dental acrylic. The contacting leads for the implant were led subcutaneously and additionally secured at the lambdoideal crista. The contact with the processor was transcutaneous in the interscapular line. At the end of the implantation, the electrical thresholds were determined using brainstem-evoked responses. The transcutaneous penetration site was covered with a jacket containing the processor. Animals were treated with ampicilline (100 mg s.c.) for 7 days post-surgery. Three days after the operation the electrical thresholds were behaviorally tested using pinna reflexes (details in Kral et al., 2002). The signal processor was activated subsequent to animal's full recovery (~7 days after implantation). The moment at which the implant was activated determined the age of onset of asymmetric hearing.

Chronic stimulation was performed using single-channel portable processors with a compressed analogue coding strategy in monopolar stimulation. Stimulation was applied continuously without interruption (on a 24/7 basis; for details see supplementary material in Kral et al., 2013). To exclude the effects of stimulation duration, three animals were stimulated for two months (~1440 h of implant stimulation), one animal for three months (~2160 h of implant stimulation) and four animals were stimulated for five months (~3600 h of implant stimulation). The animals were trained to respond to a brief tone of 732 Hz frequency by picking up a reward at a specified location on 5 days a week (20 stimulus presentations per session). The success rate exceeded 50% after 7–21 days of training in all animals and confirmed that the animals used the acoustic input to actively control behavior.

### Acute experiments: stimulation and recording

For acute experiments, all animals were premedicated with 0.25 mg atropine i.p. and initially anaesthetized with ketamin hydrochloride (24.5 mg/kg Ketavet, Parker-Davis, Germany) and propionylpromazine phosphate (2.1 mg/kg Combelen, Bayer, Germany) or xylazine hydrochloride (1 mg/kg, Bayer, Germany). The animals were then tracheotomized and artificially ventilated with 50% O_2_ and 50% N_2_O, with a 0.2–1.5% concentration of isoflurane (Lilly, Germany) added to maintain a controlled depth of anesthesia (Kral et al., 1999). It was monitored using heart-rate, end-tidal CO_2_, muscle tone and EEG signals. End-tidal CO_2_ was maintained below 4%. Core temperature was kept above 37.5°C using a homeothermic blanket. The animals' status was monitored further by blood gas concentration measurements, pH, bicarbonate concentration and base excess, glycaemia and oxygen saturation. A modified Ringer's solution containing bicarbonate (dosage based on the base excess) was infused i.v. The internal state was monitored by testing capillary blood every 12 h.

The animal's head was fixed in a stereotactic holder (Horsley-Clarke). Both bullae and ear canals were exposed. In order to record evoked auditory brainstem responses, a small trephination was drilled at the vertex and a silver-ball electrode (diameter 1 mm) was attached epidurally. Hearing status was tested at the beginning of the experiments. So as to prevent electrophonic responses, the hair cells in normal-hearing ears were destroyed by intracochlear instillation of 300 μl 2.5% neomycine sulphate solution over a 5 min. period and subsequent rinsing using Ringer's solution. The absence of hearing was subsequently confirmed by the absence of brainstem-evoked responses.

Stimulation in the final acute experiments was performed using cochlear implants inserted bilaterally into the cochleae. In chronically electrically stimulated animals, the chronic implant was used for stimulation at the “hearing” ear. The stimulus was a biphasic pulse (200 μs/phase) applied through the apicalmost electrode contact at 10 dB above the lowest cortical threshold (see Kral et al., 2009).

For recording, a trephination above the auditory cortex was performed and the dura was opened. First, within a grid of 3 × 3 positions, LFPs were recorded using low-impedance electrodes to determine the lowest cortical threshold for stimulation with a biphasic pulse (200 μs/phase) applied through the apicalmost electrode of the implant. Mapping of cortical responses was performed using glass microelectrodes (*Z* ≈ 6 MΩ) that were moved along the auditory cortex with a micromanipulator (1 μm precision) at the cortical surface. Stimulus was 10 dB above the lowest cortical thresholds, the current level at which electrically-evoked LFPs reach the saturation point. By choosing that intensity the whole active volume of the cortex can be determined. The signals were amplified 5000–10,000 times, bandpass filtered (0.01–10 kHz), digitized (at a sampling rate of 25 kHz) and 50 responses were averaged to determine the mean LFP at each recording position. First, contralateral cortex was investigated, followed by the ipsilateral cortex. The tested combinations of recording site and stimulation site are shown in Table 1.

### Data processing

**Figure 2:**
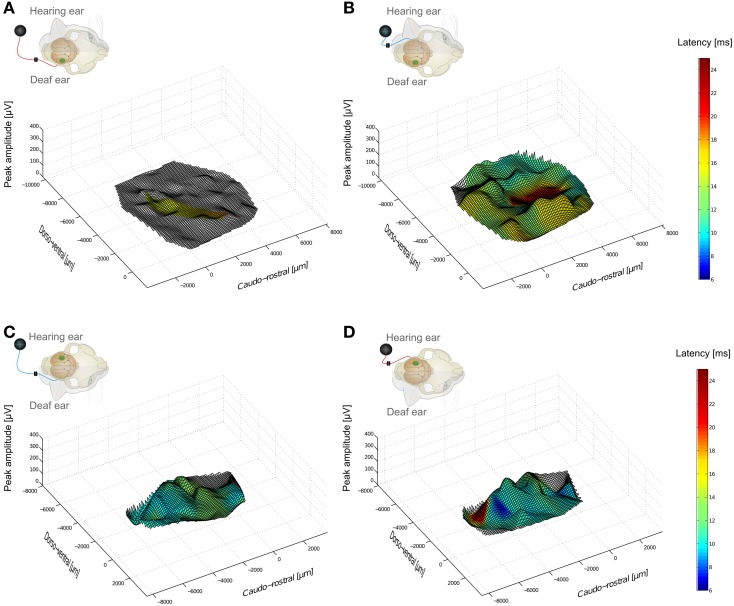
**Hemispheric specificity of amplitude effects in cortical responses of a unilaterally congenitally deaf animal investigated at the ipsilateral and contralateral cortex**. Insets indicate stimulation-recording configurations. In the inset, small loudspeaker indicates hearing side, and green spot the cortex in which recordings are made. Blue and red wires indicate ear stimulated with a cochlear implant. Largest peak amplitudes and peak latencies (color) are shown as a function of recording position. Responses below 50 μ V were not considered and are shown without coloring. **(A)** cochlear implant stimulation at the contralateral (deaf) ear, recording at hemisphere contralateral to hearing ear. Few responses above 50 μ V were observed. **(B)** in the same animal, stimulation of hearing ear extensively activated the whole contralateral primary auditory cortex. **(C,D)** at cortex ipsilateral to hearing ear, stimulation of both deaf (left) and hearing (right) ear resulted in strong activation of portions of auditory cortex.

From the more than 100 recordings at the cortical surface within field A1 and adjacent fields, cortical activation maps were constructed (Figure 2).

The electrical artifacts from the stimulation occurred between 0.0 and 0.6 ms post-stimulus and did not influence the response (latency > 7 ms). The signal before the response (500 ms duration in each animal) was characterized by computing its mean and standard deviation. The threshold of mean ± 4^*^standard deviation was then used for detecting neuronal responses. The threshold attained absolute values of 10–20 μV.

**Figure 3:**
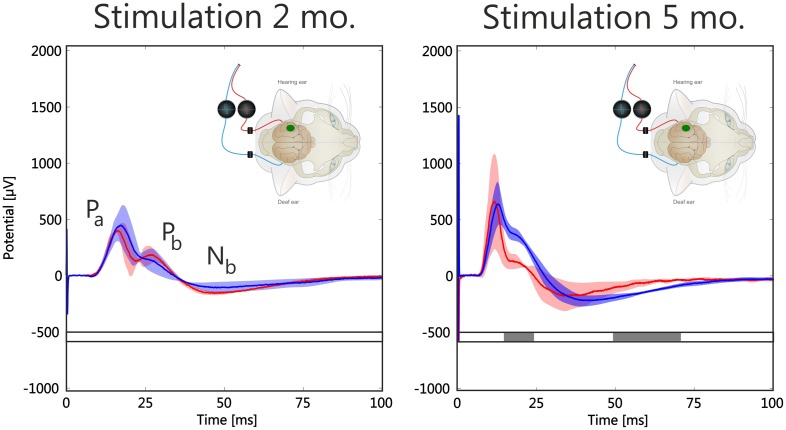
**Effect of stimulation duration (2 and 5 months) on morphology of mean local field potentials (LFPs) from hot spots in two early-implanted animals**. Inset shows configuration: recording was at the ipsilateral cortex (green spot), stimulation of hearing and deaf ear. Response color denotes stimulation site (red: uncrossed stimulation, blue: crossed stimulation); shaded area is the temporally smoothed region of one standard deviation around mean. Gray bar below curve indicates statistical significance of difference between red and blue curve using a running two-tailed Wilcoxon-Mann-Whitney test (2 ms window, α = 0.001, corrected by the false detection rate procedure). Increasing stimulation duration increased overall amplitudes of responses, but more so for hearing ear. Additionally, latencies decreased with stimulation duration. Statistically significant portions are at *P*_*b*_ and *N*_*b*_ regions.

Using this measure, onset latencies were detected for the first positive response (*P*_*a*_ component, Figure 3) of the LFPs. For each recording position of the surface maps, these data were determined for crossed and uncrossed stimulation. Normality of the data was tested using the Jarque-Bera test (5% significance level) and if confirmed, a two-tailed *t*-test was carried out; if it failed, a Wilcoxon-Mann-Whitney test (both at 5% significance level) was used. Additionally, for each position a paired difference of the onset latency was computed. The medians were used as population measures since the latency values showed a significantly skewed distribution. Comparisons between the experimental groups were performed by applying the Wilcoxon-Mann-Whitney test (two-tailed, 5% significance level).

Peak amplitudes of *P*_*a*_ components were determined using an automated procedure (based on the time derivatives of the signals). Amplitudes below 50 μV were discarded from the processing to minimize the effect of noise on small amplitude signals.

Some analyses were performed exclusively from six positions within the area with the largest responses (the “hot-spot,” Kral et al., 2009). These responses were averaged and used in comparisons. For this, a sliding window of 2 ms was used, comparisons were performed using the Wilcoxon-Mann-Whitney two-tailed *t-test* at α = 0.1%, adjusted to multiple comparisons using the false detection rate procedure (Benjamini and Hochberg, 1995).

The comparison of the morphology of mean LFP was assessed using the dissimilarity index (DI, Kral et al., 2009):

$$DI=0.5\cdot \sum_{T}|\frac{{\text{LFP}}_{1}(T)}{\sum_{t}\left|{\text{LFP}}_{1}(t)\right|}-\frac{{\text{LFP}}_{2}(T)}{\sum_{t}\left|{\text{LFP}}_{2}(t)\right|}|$$

This index considers the morphology of the LFPs irrespective of the amplitude. A dissimilarity index can reach values between 0 and 1, whereas 0 represents identical LFPs. Identical but time-reversed signals yield a high dissimilarity index due to the sample-by-sample comparison.

## Results

The present comparisons concentrated on LFP morphology and onset latency. First, the response maps and morphology of the LFPs are described, followed by onset latency comparisons.

### Terminology

In the present paper, the terms *contralateral* and *ipsilateral* are always used with reference to the side of the “chronically” hearing ear. The *hearing ear* may be either normal hearing (in unilaterally congenitally deaf animals) or born deaf and implanted at a later age (in the chronically electrically stimulated animals). The ipsilateral cortex is the one on the same side of the brain as the hearing ear.

On the other hand, the term “*crossed*” is used relative to the side of a given recording or stimulation and always refers to the “opposite” side of the brain. The term “*uncrossed*” refers to the same side as the ear stimulated or the cortex in which recordings were made.

Thus, if the left ear was the hearing ear, and a probe stimulus was presented at the right (i.e., the deaf) ear, the crossed response refers to the response recorded at the left cortex and the uncrossed response refers to that at the right cortex. In this case, the ipsilateral cortex is then the left one and the contralateral cortex is the right one.

### Spatial and temporal cortical response pattern

Crossed and uncrossed responses compared at the same cortex tend to result in different LFPs in normal hearing animals, with crossed responses showing larger amplitudes and shorter latencies (Kral et al., 2009). In binaurally deaf animals, this difference was diminished in amplitudes and absent in latencies (Kral et al., 2009). The congenitally unilaterally deaf animal exhibited a remarkable pattern of activity when the hemispheres were compared directly (Figure 2). The cortex ipsilateral to the hearing ear showed responses to both the hearing as well as the deaf ear (Kral et al., 2013). The cortex contralateral to the hearing ear, on the other hand, showed very weak responses to the stimulation of the deaf ear following congenital unilateral deafness despite good responsiveness to the hearing ear.

To minimize the effect of different spatial position when comparing responses at the contralateral and the ipsilateral cortex, further analysis was concentrated on hot spots: the areas of largest responses. These were considered as being the cortical representation of the stimulated region in the cochlea and were therefore considered the functionally corresponding sites in the cortex. From a total area of 1.5 mm^2^ within the hot spot, all LFPs were averaged (~6 recording positions; Figure 3).

First, the effect of stimulation duration was compared in early-implanted animals (Figure 3, animals 3 and 4, Table 1): Longer experience with a unilateral cochlear implant increased the amplitudes of the responses in the hot spot, and more so for the hearing ear; however, responses to both the hearing and the deaf ear increased. At longer latencies (component *P*_*b*_ and later), more significant differences for the two stimulation sites were observed than at short latencies (Figure 3; see also below).

**Figure 4:**
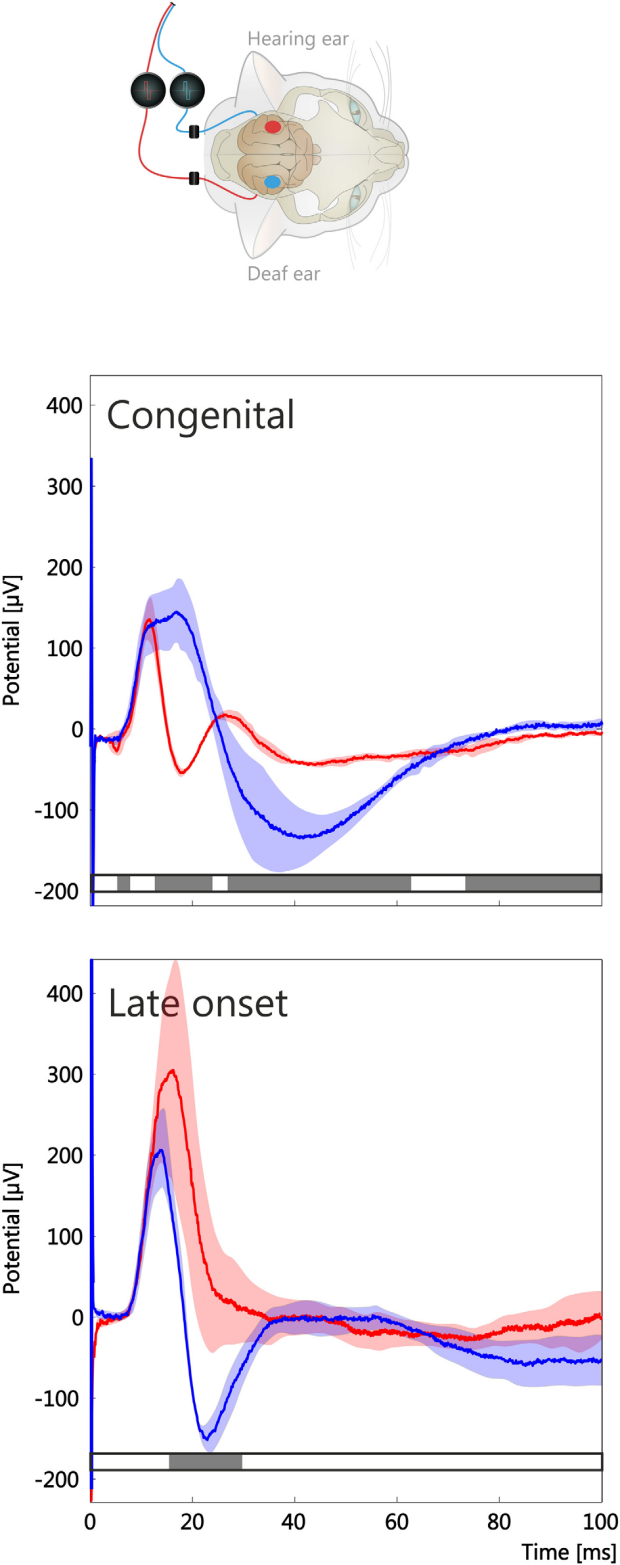
**Crossed responses in a congenitally unilaterally deaf animal and a late-implanted animal (stimulation duration 5 months)**. Stimulation of hearing ear and recording at contralateral cortex shown in blue, for deaf ear and recording at the ipsilateral hemisphere in red, shaded area is the temporally smoothed region of one standard deviation around mean. Gray bars below curve indicate statistical significance of a running two-tailed Wilcoxon-Mann-Whitney test (α = 0.001, corrected by false detection rate procedure, 2 ms window). Ear-specific effects were observed in both animals; however, regions of statistical significance were larger in the congenital onset case. Maximum amplitudes in both compared animals were within 200–400 μV, as reported previously (Kral et al., 2009).

Crossed responses to stimulation of the deaf ear and to stimulation of the hearing ear were compared next (Figure 4). For crossed responses the maximum amplitudes were of a similar order of magnitude for both ears, although significant interindividual variability was noted in the maximum potential. However, in the congenitally unilaterally deaf animal, a marked difference in the morphology of the response was observed at longer latencies (Figure 4, top). In particular, component *P*_*b*_ was well separated from the *P*_*a*_ component when the deaf ear was stimulated (Figure 4 top, red curve), whereas the components appeared fused when the hearing ear was stimulated (Figure 4 top, blue curve). A morphological difference of this nature was less expressed in late-implanted animals, particularly since *P*_*b*_ could not always be identified (Figure 4, lower panel).

**Figure 5:**
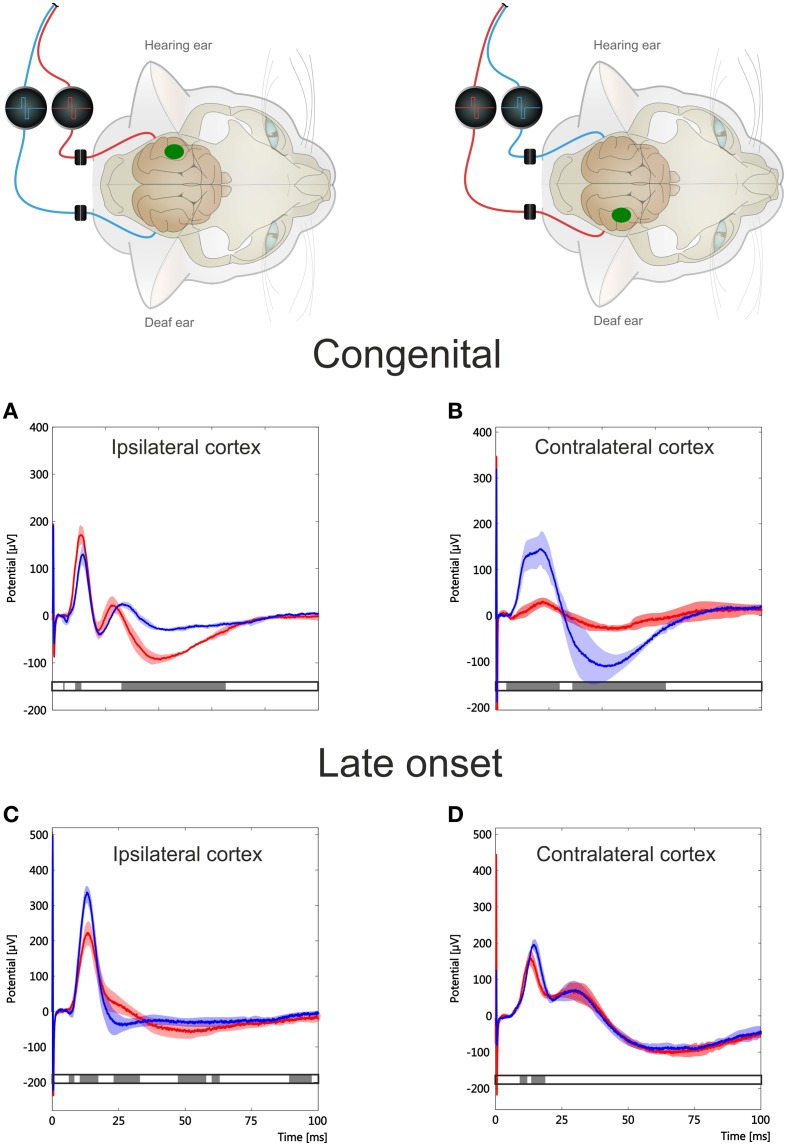
**Comparison of crossed and uncrossed responses at both hemispheres in a congenitally unilaterally deaf animal (A,B) and a late implanted animal (C,D)**. Inset shows stimulus configuration; green spot shows recorded hemisphere. Crossed response is denoted in blue, uncrossed response in red; shaded area is the temporally smoothed region of one standard deviation around mean. Gray bar below curve indicates statistical significance of a running two-tailed Wilcoxon-Mann-Whitney test (α = 0.001, corrected by false detection rate procedure, 2 ms window). Morphology of LFPs shows temporal regions of significant difference in all configurations, but more so in the congenitally unilaterally deaf animal. Further, morphology is more different between hemispheres than within one hemisphere. Note differences in ordinate. Early-implanted animals had larger maximal amplitudes than congenital unilateral and late-implanted animals. The absolute LFP amplitude showed a high interindividual variability.

At the ipsilateral hemisphere early-implanted animals had uncrossed responses larger than crossed ones (Figure 5A). This was not observed in the late-implanted animals (Figure 5C, comp. Kral et al., 2013). The crossed and uncrossed mean response had similar morphology when considered at the same hemisphere (comparisons within the panels A–D in Figure 5). At the cortex contralateral to the hearing ear, a marked difference in amplitude of the crossed and uncrossed response was evident in the congenitally unilaterally deaf animal (Figure 5B; see also Figure 2). Nonetheless, even in this case it was apparent that the LFP morphology within a hemisphere was more similar than between hemispheres. In total, the responses at the same hemisphere (comparisons within panels Figures 5A–D) were more similar than those compared between hemispheres (comparisons between panels in Figures 5A,B or Figures 5C,D), irrespective of the age of onset.

**Figure 6:**
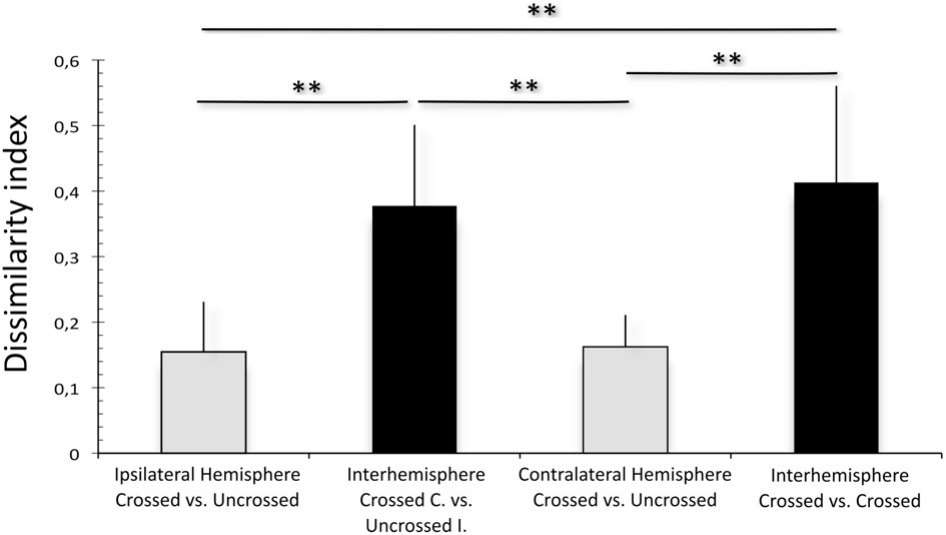
**Dissimilarity index computed between mean LFPs within one hemisphere (gray) and between hemispheres (black)**. Interhemisphere comparisons result in significantly higher dissimilarity index than within-hemisphere comparisons. Two-tailed Wilcoxon-Mann-Whitney test, ^**^p < 0.01.

To quantify this observation, dissimilarity index for *P*_*a*_/*P*_*b*_ complex was computed for different intrahemispheric and interhemispheric comparisons (Figure 6). A Kruskal-Wallis test demonstrated a significant effect of the configuration (*p* = 0.01). For the crossed and uncrossed responses at the ipsilateral hemisphere, the LFP morphology was similar (a small *DI* of 0.16 ± 0.08, *n* = 6). However, comparing the crossed response of the contralateral hemisphere to the uncrossed response of the ipsilateral hemisphere resulted in larger difference (same animals, *DI* = 0.38 ± 0.12, two-tailed Wilcoxon-Mann-Whitney test, *p* = 0.010). Further, crossed responses (compared between hemispheres) showed a high dissimilarity index of similar magnitude as the latter comparison (*DI* = 0.41 ± 0.15, *p* = 0.749). The dissimilarity index at the contralateral hemisphere for the crossed vs. uncrossed comparison was not different from the same at the ipsilateral hemisphere (*DI* = 0.16 ± 0.08 vs. 0.16 ± 0.05, *p* = 1.000). Thus, interhemispheric comparisons resulted in larger *DI*s than intrahemispheric comparisons. Taken together, these results demonstrate that the cortical response shows hemispheric specificity in morphology irrespective of the ear that is stimulated. Although the largest *DI*s were found in the congenitally unilaterally deaf animal (Figures 5A,B), the correlation with onset age was not significant in *DI* measures (α = 5%).

### Onset latencies

A previous study demonstrated high plasticity of LFP onset latency and its sensitivity to developmental modifications (Kral et al., 2013). This measure showed lower variance over cortical positions and animal than peak amplitudes, so that interhemispheric comparisons were statistically testable and recording positions biased less to the comparisons. For these reasons, further comparisons were performed using onset latencies of LFPs. In the previous study (Kral et al., 2013), we performed paired comparisons at the ipsilateral cortex; in the present study, we had to perform unpaired comparisons (between hemispheres and animals).

**Figure 7:**
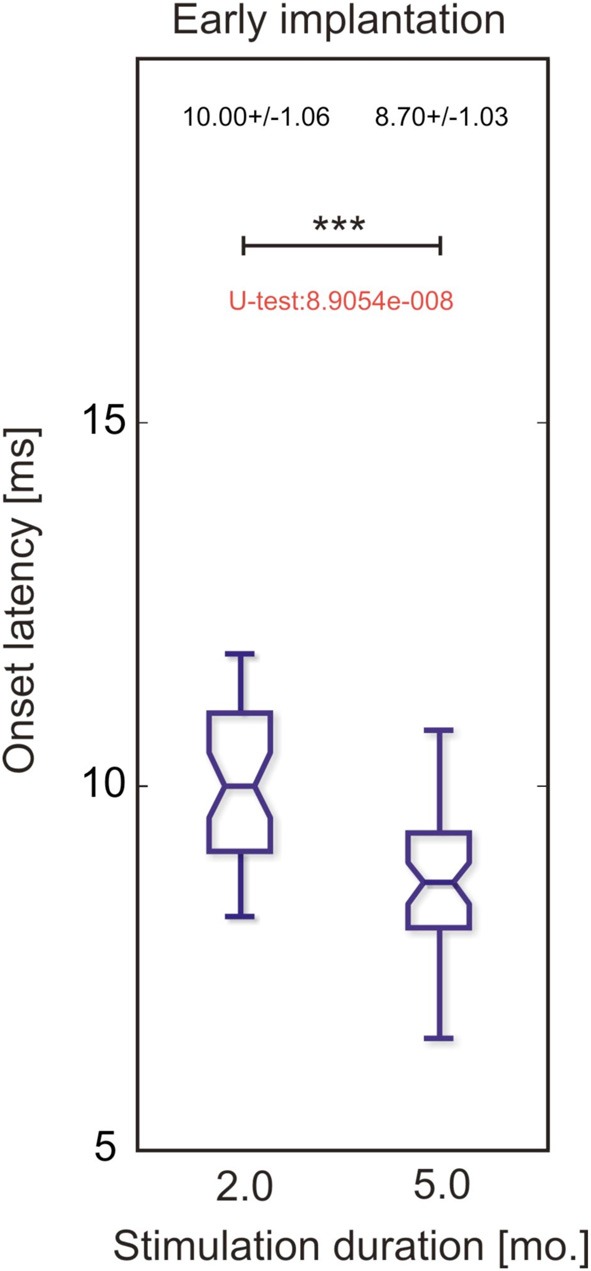
**Onset latency at the cortex contralateral to hearing ear in response to stimulation of hearing ear in two animals implanted at 3.5 months**. One was stimulated for 2 months and the other for 5 months. Longer stimulation resulted in shorter-onset latency (two-tailed Wilcoxon-Mann-Whitney test, ^***^~*p* < 0.001).

First, the effect of stimulation duration and implantation age on onset latencies of crossed responses was determined at the contralateral hemisphere for stimulation of the hearing ear (Figure 7). In animals implanted early (3.5 months), as expected, stimulation duration was associated with a decrease in median onset latency, demonstrating that onset latency does change as a result of the stimulation. Moreover, increasing implantation age decreased the effect of stimulation on contralateral onset latency after 5 months of chronic electrical stimulation, changing from 9.1 ± 1.89 ms (median ± absolute deviation of the median) at 3.5 months to 9.95 ± 1.00 ms at 5.0 months to finally reach 10.5 ± 2.63 at 6 months onset (significant increases with increasing age of onset, two-tailed Wilcoxon-Mann-Whitney test at α = 0.05). Therefore, onset latency reflects the amount of hearing experience and age of onset and thus represents a good measure of plastic adaptation caused by the stimulation.

**Figure 8:**
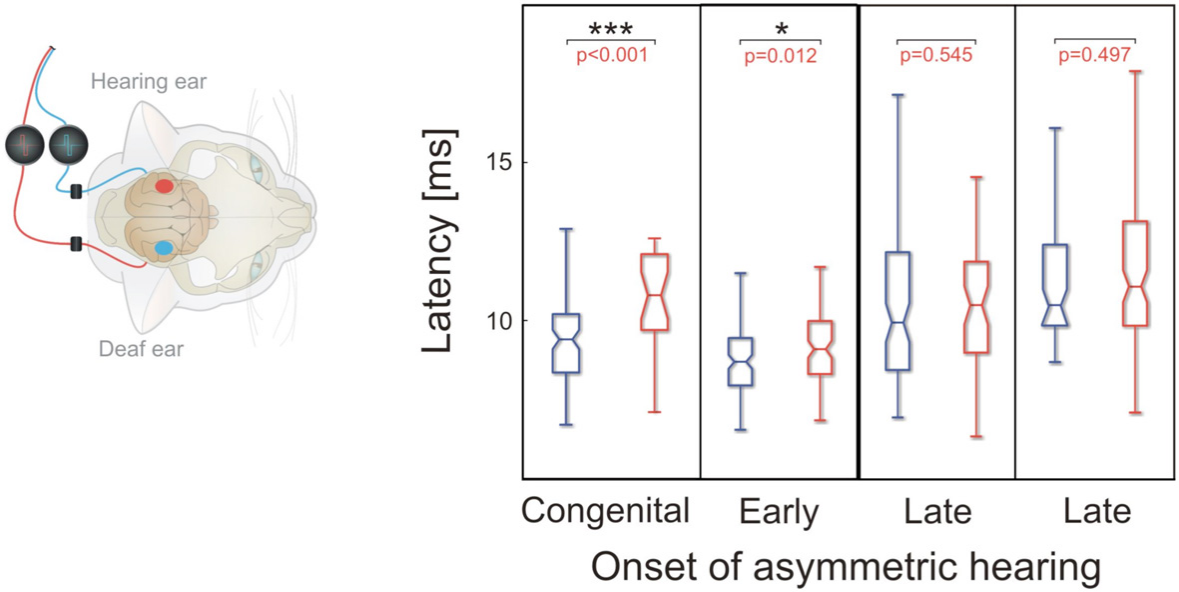
**Onset latency for crossed responses; color denotes configuration**. Blue denotes the hearing ear. In animals with early onset of unilateral hearing, crossed response for hearing ear was significantly shorter than that for the deaf ear (two-tailed Wilcoxon-Mann-Whitney test). This was not the case for late-onset animals. Onset latency for stimulation of deaf ear did not show any systematic dependence on onset of asymmetric hearing, whereas onset latency for hearing ear became longer in cases of late-onset asymmetric hearing. ^***^~*p* < 0.001; ^*^~*p* < 0.05.

Next, onset latencies of crossed responses were compared between hemispheres in four animals with a stimulation duration of 5 months (Figure 8). Here, stimulation of the hearing ear resulted in shorter crossed response latencies than that of the deaf ear. The effect was significant in early onset animals and disappeared in late-implanted animals. However, when considering the crossed pathway of the deaf ear, the latency of the congenital and the late implanted animals were similar and we could not detect any consistent age-of-onset effect. Consequently, with regard to onset latency, the effect of stimulation in crossed responses is confined to the hearing ear; the influence on the crossed response of the deaf ear was weak. Remember that the amplitudes were, however, similar for the crossed response of the deaf and the hearing ear (Figure 4).

**Figure 9:**
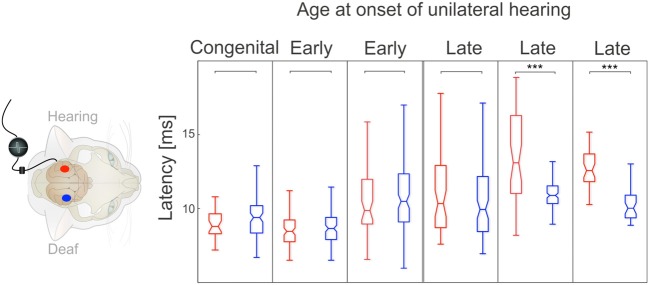
**Comparison of crossed and uncrossed responses for hearing ear**. Color denotes hemisphere in which recordings are made (red: ipsilateral, blue: contralateral). In all early-onset animals, ipsilateral hemisphere showed a nominally smaller median onset latency, but difference was not statistically significant (two-tailed Wilcoxon-Mann-Whitney test). In all late-onset animals, contralateral hemisphere showed a smaller median latency, but in only two of three animals was the difference statistically significant (two-tailed Wilcoxon-Mann-Whitney test). Note that both ipsilateral and contralateral latencies were greater in late-onset animals. ^***^~*p* < 0.001.

Finally, the onset latencies for the responses to the stimulated (hearing) ear were compared between the cortices (Figure 9). This comparison was performed on one congenitally unilaterally deaf and five implanted animals, two with early and three with late implantation. In the animals with early onset of asymmetric hearing, the latencies were short and not different at the contralateral and ipsilateral cortex, with a tendency toward shorter latencies for ipsilateral (uncrossed) response. This is highly unusual, as in all hearing cats the situation was the reverse and significant (Kral et al., 2013). However, in late-implanted animals, the latencies tended to be longer (see Figure 9), and in two out of three animals tested, contralateral onset latency was significantly smaller than the ipsilateral onset latency (in the remaining animal it was not significant, but with a tendency toward shorter contralateral response, Figure 9). That demonstrates that in the late implantation, there is some profit from the stimulation, but it is more confined to the crossed response. In early implantation, there is benefit to both the uncrossed and crossed response. In this respect, the afferent pathway to the contralateral cortex has a longer sensitive period than the one to the ipsilateral cortex.

**Figure 10:**
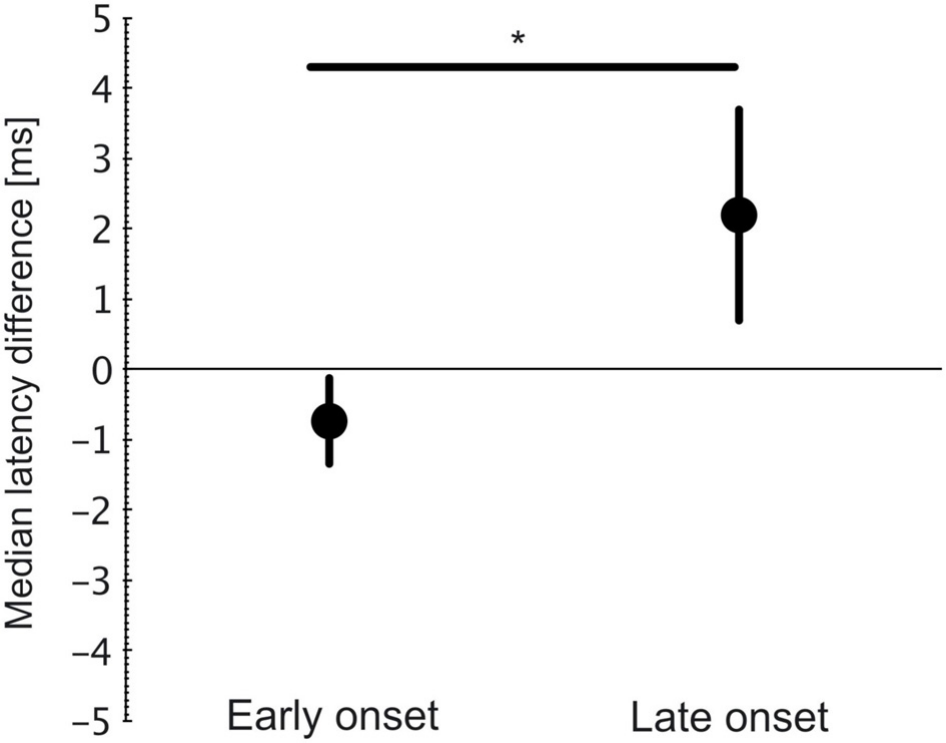
**Difference between median latencies (uncrossed–crossed response) for hearing ear**. All three early-onset animals had negative differences (uncrossed < crossed), whereas all late-onset animals had positive differences (uncrossed > crossed). Two-tailed Wilcoxon-Mann-Whitney test, *p* = 0.04 (^*^~*p* < 0.05).

To verify this outcome, the difference in medians of uncrossed and crossed responses to the hearing ear was compared between early and late onset of asymmetric hearing (Figure 10). In early-onset animals, uncrossed response showed a shorter onset latency, resulting in a negative mean difference in all three animals, but the difference became positive in all three cases of late implantations (Wilcoxon-Mann-Whitney two-tailed test, *p* = 0.044, Figure 10). This further demonstrates that the benefit to the uncrossed response is confined to early-onset asymmetric hearing.

## Discussion

The present manuscript describes hemispheric specificity of effects of unilateral hearing following congenital deafness. It demonstrates that in early-onset unilateral hearing, both the ipsilateral and the contralateral hemisphere reorganize and strengthen the responses to stimulation of the hearing ear, giving it an advantage over the deaf ear. In the early-onset animals, the ipsilateral hemisphere responded more strongly to stimulation of the hearing ear. The morphology of the LFPs demonstrated that the reorganizations following unilateral deafness were hemisphere-specific (Figure 6).

The uncrossed pathway appeared more susceptible to developmental alteration of hearing balance than the crossed pathway, although both the crossed and uncrossed responses to the hearing ear were changed by the stimulation. In late implantations, the uncrossed response did not show similar reorganization, and it was largely the crossed pathway of the hearing ear that still benefited from stimulation, although less than in early implanted animals (Figures 8, 9). The uncrossed pathway was more sensitive to age of onset than was the crossed pathway, demonstrating a shorter sensitive period. The mutual relationship between the hearing ear and the hemisphere investigated is critically important when assessing developmental auditory plasticity.

### Methodological discussion

The present study compared the effects of stimulation at both hemispheres. There are several limitations to the present approach that merit discussion. First of all, comparisons between hemispheres preclude pairwise testing. Although the present study used an approach validated by several previous studies performing interindividual comparisons (Klinke et al., 1999; Kral et al., 2002, 2006), the present approach is more limited than the pairwise comparison (Kral et al., 2013). The present experiments took 48 h in most animals, with the possibility of a state change during the procedure. However, even though recordings at the ipsilateral cortex were performed later, the responses to stimulation were not systematically different in hemispheres exposed later than those exposed earlier (e.g., for the hearing ear or the amplitudes of crossed responses; see examples in Figures 2, 4 and 5). Furthermore, onset latencies systematically changed depending on experience and on which ear was stimulated, and not on time of recording (later-exposed cortices showed shorter onset latencies in early-implanted animals and longer ones in the late-implanted animals), so that a change of state during the experiments can be ruled out. In total, we have no indication of any systematic shifts in the general state of the animals during recordings.

Degeneration of spiral ganglion cells is unlikely to have contributed to the findings here, as there was no significant spiral ganglion cell loss in the implanted (basal) region of the cochlea within the first 2 years of life in congenitally deaf cats, and even at an older age there was less degeneration in the basal cochlea (Heid et al., 1998). Moreover, stimulation of the deaf ear in unilateral animals resulted in much smaller responses at the hemisphere contralateral to the hearing ear, but at the ipsilateral cortex the responses to the deaf ear were comparable to the responses to the hearing ear (Figures 2, 6), showing not only the hemispheric specificity of plastic reorganization, but also ruling out any significant influence of peripheral ear-specific effects.

Finally, age at final experiment did not significantly contribute to the present findings. The cortical developmental sequence in deaf and hearing cats with respect to electrically evoked responses terminates at ~6 months (Kral et al., 2005; for similar data on acoustic stimulation, see Eggermont, 1996; for data on onset latencies, see supplementary material in Kral et al., 2013). Only two of the 10 investigated animals were younger (animals no. 3 and 6 in Table 1). Omitting these two animals from the comparisons did not affect any finding of the study. Overall, we can also rule out age at experiment as a confounding factor.

Finally, it has to be considered that the effects measured at the ipsilateral cortex need not necessarily arise in the ipsilateral hemisphere, but may have an origin in the contralateral hemisphere before pathway crossing. For the sake of simplicity, however, we will not complicate the considerations below by including this aspect.

### Discussion of results

The present study is well in agreement with previous investigations on the subject. The notion of auditory sensitive periods in neuronal plasticity (review in Kral, 2013) has been established for deaf, cochlear-implanted animals (Kral et al., 2001, 2002, 2013) and cochlear-implanted children (Ponton and Eggermont, 2001; Sharma et al., 2002, 2005), as well as for hearing animals (Zhang et al., 2002; Nakahara et al., 2004; de Villers-Sidani et al., 2008). However, sensitive periods are not observed in all plastic reorganizations in the brain (Noreña et al., 2006; Eggermont, 2013; Pienkowski et al., 2013).

Early neonatal unilateral ablation studies suggested a reorganization of the auditory brain toward the hearing ear (Nordeen et al., 1983; Kitzes and Semple, 1985), although it has not been possible to stimulate the ablated ear and compared it with the hearing ear, and no developmental study has been undertaken.

The reorganization reported here and in previous studies is in accord with results from cochlear-implanted children (Peters et al., 2007; Zeitler et al., 2008; Graham et al., 2009; Gordon et al., 2013; Illg et al., 2013). Early second implantations are important in terms of retaining the potential to reverse the aural preference, whereas the effects were observed after more than 1 year of unilateral use (in early implantations at 1.74 years, Gordon et al., 2013; in single-sided deafness, see Scheffler et al., 1998; Bilecen et al., 2000; Langers et al., 2005; Burton et al., 2012; Maslin et al., 2013). As the greatest effects were observed in congenitally unilaterally deaf animals in the present study, before cortical synaptogenesis has set in in cats (Kral et al., 2013), implantation at ages of less than 1 year of life (peak of synaptogenesis between 1 and 4 years, comparison cat-human in Kral and O'Donoghue, 2010) can be expected to generate substantially larger effects in children than those described to date. It has to be stressed here, however, that although age at first implantation is important for the outcome in pediatric cochlear implantation, some benefit from the second ear is found even in cases of longer interimplant delay (Zeitler et al., 2008; Illg et al., 2013). This corresponds to the present observation that in no case was the response to the deaf ear eliminated completely (see also Kral et al., 2013). Nonetheless, lesser responses for the deaf ear at the contralateral cortex supports the suggestion that the deaf ear is placed at a disadvantage in competition for cortical resources particularly in individuals with early onset-unilateral hearing (Kral et al., 2013).

Importantly, the outcome shows specificity for the ear that has received input (Figures 2, 4, 8). Both onset latency (Figure 8) and amplitudes (Kral et al., 2013) of the responses appeared different for different ears, but a more detailed analysis demonstrated that the morphology (disregarding amplitudes) is more hemispheric-specific than ear-specific (Figures 5, 6). Nonetheless, a lesser response was observed for the deaf ear, particularly at the contralateral hemisphere in the congenital animal (Figure 2). At the ipsilateral hemisphere, a pairwise comparison demonstrated smaller responses for the deaf ear (Kral et al., 2013). Pairwise testing was unfortunately not possible for the present interhemispheric comparisons, so that small differences may went unnoticed.

Subcortical reorganization with deafness and cochlear implants has been described before (Snyder et al., 1991; Shepherd et al., 1999; Ryugo et al., 2005; O'Neil et al., 2010). Nevertheless, several studies indicate that cortical plasticity is higher than subcortical (Ma and Suga, 2005; Popescu and Polley, 2010), and that the former plays the controlling role via efferent systems (Ma and Suga, 2005). Interestingly, despite pronounced developmental effects observed in other measures, the dissimilarity index did not show a clear developmental pattern. For the present experiments, this means that it is more the balance of aural inputs that is developmentally modulated and less the way in which the inputs are processed after converging on the same neuronal elements. Further it shows that the plasticity mechanism is similar at all ages, only the extent of the effect fades with increasing age, and fades out faster at the ipsilateral hemisphere. The present study indicates a subcortical site for reorganization following unilateral deafness (before or at the point of binaural convergence).

The uncrossed response latency for stimulation of the hearing ear became smaller than the crossed response latency, so that the difference in medians was negative, but only in the early-implanted animals (Figures 9, 10). This finding could indicate a loss of inhibition at the ipsilateral cortex in early-implanted animals, allowing uncrossed inputs to be pulled to much shorter latency (Zhou et al., 2012). In normal, binaural hearing animals, the uncrossed response evokes inhibition more frequently, whereas the crossed response is more excitatory (Zhang et al., 2004). The present observations can therefore be explained by a specific down-regulation of inhibition in unilateral hearing at the hemisphere ipsilateral to the hearing ear (Vale et al., 2004), as it may explain the shorter latency, larger uncrossed response for the hearing ear, as compared with the longer-latency, smaller uncrossed response for the deaf ear. Different recruitment of inhibition in the ipsilateral and contralateral hemisphere would then also affect the morphology of the LFPs differentially for the different hemispheres. The relatively large drop in onset latency in early-onset animals (uncrossed response in the ipsilateral hemisphere) can be alternatively explained only by an increase in synaptic conduction in many synapses; this, however, fails to explain the hemispheric specificity of the outcomes on LFP morphology. Finally, a downregulation of inhibition in (preferentially) the ipsilateral hemisphere can explain the rapid onset of the effect that is different from the process behind the slower plastic changes in the contralateral hemisphere (Figure 7, see also Kral et al., 2002). Previous experiments with unilateral cochlear ablation also suggest that the mechanism underlying ipsilateral adaptations in the midbrain differs from that underlying contralateral adaptations (Vale et al., 2004). Future studies on unit activity in congenitally unilaterally deaf and unilaterally implanted animals may demonstrate this by showing the reduction or absence of suppressive binaural interaction in the ipsilateral hemisphere and its presence in the contralateral hemisphere.

Inhibitory synaptic transmission matures later than excitation (review in Kral et al., 2013) and is likely one of the mechanisms explaining the shorter sensitive period for the uncrossed response. In this sense the longer onset latency with increasing implantation age at the ipsilateral hemisphere could be related to the fact that in early-implanted animals, the developmental process of inhibitory transmission was not yet finalized when the hearing asymmetry started and could be therefore more modulated by unilateral hearing. In late-implanted animals the unilateral hearing set in after the development of inhibition has terminated. Plasticity observed before this point is likely to involve changes on both inhibitory and excitatory transmission, whereas later plasticity likely depends more on excitatory synapses with smaller contribution of inhibitory synapses. That can explain the different sensitive periods at the ipsilateral and contralateral hemisphere.

**Figure 11:**
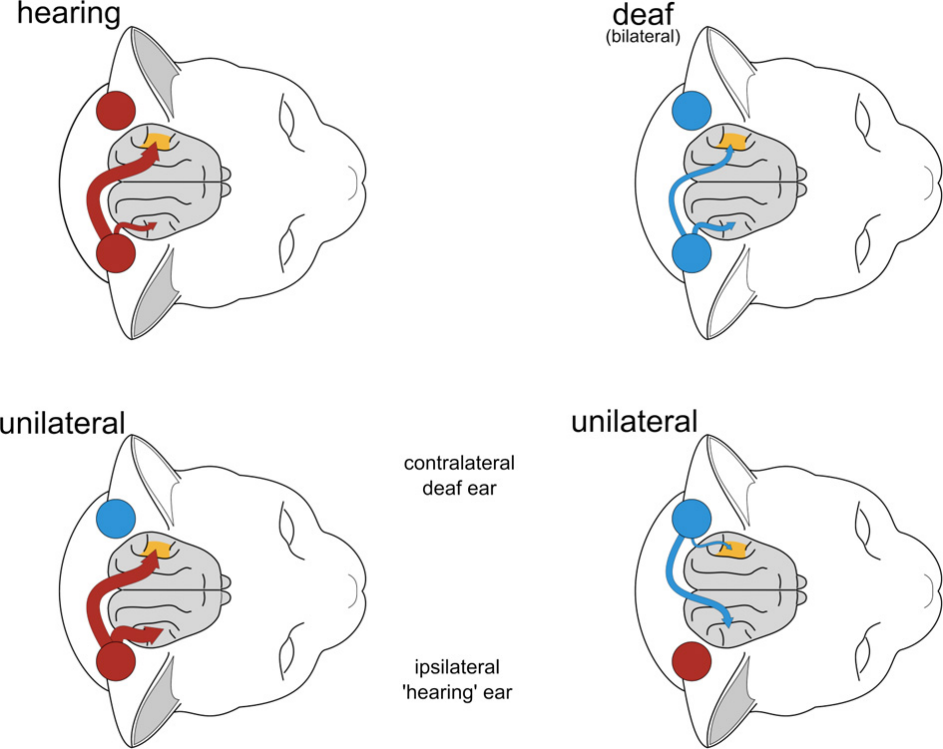
**Graphical summary of results from this and two previous studies (Kral et al., 2009, 2013)**. In binaurally deaf animals, contralaterality was reduced. In early-onset unilaterally deaf animals, hearing ear resulted in reduced contralaterality (when assessed from crossed and uncrossed response for hearing ear), whereas deaf ear showed increased contralaterality (when assessed from crossed and uncrossed response for deaf ear) due to reduced uncrossed response.

Stimulation of the hearing ear generates strong responses both at the ipsilateral and the contralateral hemisphere (for human data, see Bilecen et al., 2000; Hanss et al., 2009; Burton et al., 2012; Gordon et al., 2013). Stimulation of the deaf ear activates the crossed pathway but does so only weakly for the uncrossed pathway. The contralateral preference of the deaf ear is strengthened due to reduction of the uncrossed responses, the contralateral preference of the hearing ear being reduced because of to strengthening of the uncrossed responses (Figure 11). The present findings therefore provide an explanation of the mechanism behind the outcomes of human imaging studies (Bilecen et al., 2000; Gordon et al., 2013).

## Conclusion

The present study supports the concept of several sensitive developmental periods by demonstrating a shorter sensitive period for reorganization at the ipsilateral hemisphere as compared with the contralateral hemisphere. It shows more extensive changes in uncrossed responses than in the crossed responses in early-onset animals. Furthermore, it shows that unilateral deafness results in an asymmetric brain, with different hemispheres showing differential responses for both the deaf and the hearing ear. The hemisphere ipsilateral to the hearing ear most likely downregulates inhibition, by that specifically decreasing onset latency of the response to the hearing ear. This effect is not found in the contralateral hemisphere.

The deaf ear is, however, not completely ‘disconnected’ from the cortex following single-sided deafness. The hemisphere ipsilateral to the hearing ear preserves responsiveness to the deaf ear, although with a preference for the hearing ear. Finally, the present results support a greater ‘separation’ of the ears in early onset unilateral hearing.

### Conflict of interest statement

Dr. Jochen Tillein works also for MedEl Company, Innsbruck. His obligations in the company had no interference with the work nor is there any direct financial interaction between MedEl and the research performed in this study. The other authors declare that the research was conducted in the absence of any commercial or financial relationships that could be construed as a potential conflict of interest.
